# A cross-sectional and longitudinal study on the protective effect of bilingualism against dementia using brain atrophy and cognitive measures

**DOI:** 10.1186/s13195-020-0581-1

**Published:** 2020-01-10

**Authors:** Víctor Costumero, Lidon Marin-Marin, Marco Calabria, Vicente Belloch, Joaquín Escudero, Miguel Baquero, Mireia Hernandez, Juan Ruiz de Miras, Albert Costa, Maria-Antònia Parcet, César Ávila

**Affiliations:** 1 Center for Brain and Cognition, University Pompeu Fabra, Barcelona, Spain; 2 Neuropsychology and Functional Neuroimaging Group, University Jaume I, Castellón, Spain; 3 ERI Lectura, University of Valencia, Valencia, Spain; 4 ERESA Medical Group, Valencia, Spain; 5 Department of Neurology, General Hospital of Valencia, Valencia, Spain; 6 Neurology Unit, University and Polytechnic Hospital La Fe, Valencia, Spain; 7 Department of Cognition, Development, and Educational Psychology Section of Cognitive Processes, University of Barcelona, Barcelona, Spain; 8 Cognition and Brain Plasticity Group, Bellvitge Biomedical Research Institute (IDIBELL), L’Hospitalet de Llobregat, Spain; 9 Computer Science Department, University of Jaén, Jaén, Spain

**Keywords:** Bilingualism, Cognitive reserve, Alzheimer’s disease, Mild cognitive impairment, Brain atrophy, Region-based morphometry

## Abstract

**Background:**

Evidence from previous studies suggests that bilingualism contributes to cognitive reserve because bilinguals manifest the first symptoms of Alzheimer’s disease (AD) up to 5 years later than monolinguals. Other cross-sectional studies demonstrate that bilinguals show greater amounts of brain atrophy and hypometabolism than monolinguals, despite sharing the same diagnosis and suffering from the same symptoms. However, these studies may be biased by possible pre-existing between-group differences.

**Methods:**

In this study, we used global parenchymal measures of atrophy and cognitive tests to investigate the protective effect of bilingualism against dementia cross-sectionally and prospectively, using a sample of bilinguals and monolinguals in the same clinical stage and matched on sociodemographic variables.

**Results:**

Our results suggest that the two groups did not differ in their cognitive status at baseline, but bilinguals had less parenchymal volume than monolinguals, especially in areas related to brain atrophy in dementia. In addition, a longitudinal prospective analysis revealed that monolinguals lost more parenchyma and had more cognitive decline than bilinguals in a mean follow-up period of 7 months.

**Conclusion:**

These results provide the first prospective evidence that bilingualism may act as a neuroprotective factor against dementia and could be considered a factor in cognitive reserve.

## Background

The continuous use of two languages has been shown to be among the different social, physical, mental, and leisure activities [1] that can promote cognitive reserve (CR) [2–4]. CR refers to individual differences in clinical resilience to brain pathology as a result of differences in neural efficiency and/or the use of a compensatory neural network [5, 6]. This relationship between CR and bilingualism has often been related to the supposed cognitive benefits of having to manage two languages—a phenomenon, however, that is currently under dispute [7–10].

Most of the evidence supporting the potential effect of bilingualism on CR comes from studies with brain-damaged patients with cognitive decline or dementia (Alzheimer’s disease (AD), single-domain amnestic mild cognitive impairment (MCI) patients, or other patients with dementias, such as the vascular type). In this regard, the most consistent finding is that the onset of the clinical symptoms associated with the disease is significantly delayed in bilinguals, compared to monolinguals—a delay of about 4–5 years [3, 9, 11–16]. Moreover, protective effects of bilingualism against age-related cognitive decline have been found independently of baseline cognitive ability (childhood intelligence), therefore dismissing reverse causality—the fact that childhood differences such as intelligence could lead to bilingualism instead of bilingualism leading to cognitive differences [17]. However, these results have not always been replicated [4, 18].

The delay in the onset of dementia in bilinguals has been proposed as evidence of the contribution of bilingualism to CR [9]. Two brain mechanisms are suggested to explain the increased CR: neural reserve and neural compensation. Neural reserve addresses the idea that CR could be associated with individual differences in the resilience of pre-existing cognitive networks [9, 19]. In this regard, evidence from neuroanatomic studies with healthy older participants suggests that bilingualism promotes gray matter volume and white matter integrity as a result of using two languages [20–22]. Neural compensation appears when cognitive function is maintained in the presence of brain atrophy due to better utilization of alternative networks [9, 19, 23]. It has been suggested that this compensation promotes brain reserve; that is, it increases brain size in specific areas that allow more plasticity to overcome pathology and neurological insult [23].

This study takes this perspective and investigates brain atrophy in bilingual and monolingual individuals with MCI. Therefore, if bilingualism promotes neural reserve and compensation, bilinguals might be expected to tolerate greater amounts of neuropathology or atrophy once the disease is manifested. This prediction is based on neuroimaging studies showing that, at the same cognitive level, bilinguals have more brain pathology than monolinguals. The first study to investigate the CR effects of bilingualism showed that bilingual patients suffering from AD exhibited much greater brain atrophy than monolinguals in regions associated with the pathology, such as the left middle temporal lobes [24]. Because the two language groups showed the same degree of cognitive decline, the interpretation is that bilinguals would tolerate greater amounts of neuropathology or atrophy before the disease is manifested. Consistent with this interpretation, bilinguals with AD also showed greater levels of hypometabolism in the left parietal, temporal, and frontal areas than monolinguals [16]. However, it is worth noting that the opposite results have been reported in MCI patients [25].

The second objective of the study is to prospectively investigate the atrophy rate in MCI participants. Although the number of studies is limited, there is evidence suggesting that bilingualism promotes neural compensation. On the one hand, studies in healthy older individuals and patients with Alzheimer’s disease have shown increased functional connectivity in bilinguals compared to monolinguals [16, 26]. On the other hand, evidence from studies investigating structural differences in healthy older participants suggests that bilingualism contributes to brain reserve, by showing that monolinguals present more extended age-related brain atrophy than bilinguals in diverse areas of the frontal, parietal, and temporal lobules and that these differences are associated with cognitive performance on different kinds of tasks [27–30]. Together, these results support the view that bilingualism contributes to neural compensation and brain reserve during all the stages of neurodegeneration. However, this interpretation is limited by the fact that all these findings stem from cross-sectional designs that could be biased by cohort effects [31].

In the present study, we adopt both a cross-sectional and a longitudinal perspective to determine the role of bilingualism in brain atrophy and cognitive decline. Based on the reviewed literature, we hypothesize the following: (1) MCI bilinguals matched with MCI monolinguals on a cognitive level, and sociodemographic factors would transversally present greater brain atrophy; (2) MCI bilinguals would show less atrophy and cognitive decline than monolinguals longitudinally. By combining the cross-sectional and longitudinal data, we attempt to provide a plausible explanation for the nature and origin of the bilingual delay in the onset of dementia.

## Methods

### Participants

Ninety-nine MCI individuals were included in this study (49 women; mean age = 73.9 ± 5.8). All of them were born in Spain and recruited from dementia units of the Valencian community public healthcare system, and they met the following inclusion criteria: (1) subjective memory complaints (self-reported or confirmed by an informant), (2) objective memory impairment assessed with the logical memory subtest II of the Wechsler Memory Scale-III (WMS-III) [32], (3) essentially intact activities in daily living, (4) no evidence of dementia, and (5) a Clinical Dementia Rating score of 0.5. Exclusion criteria were having the following: (1) other nervous system diseases, such as a brain tumor, cerebrovascular disease, encephalitis, or epilepsy, or meeting the criteria for dementia; (2) a Geriatric Depression Scale [33, 34] score > 6; (3) visible cerebral abnormalities reported by a radiologist with experience in magnetic resonance images, such as leukoaraiosis and infarction; and (4) a current psychiatric disorder or use of psychoactive medication.

### Language group formation

All the participants resided permanently in the Spanish region of Valencia. During a clinical interview, language history was assessed using a short interview with the patient and some relatives. We asked for relevant information about three issues: (1) Age of acquisition of Catalan and Spanish; (2) self-rating of language proficiency, including their speaking and comprehension; and (3) language use based on the frequency with which they currently speak each of the two languages. Participants who reported Catalan as their mother tongue, Spanish as a second language learned at school, and active use of both languages were considered bilinguals (*n* = 39), whereas those who only spoke Spanish were considered monolinguals (*n* = 60). All the bilinguals learned Catalan at home before starting school and used it in their daily lives, but they also spoke Spanish frequently. In the area where this study was carried out, 60% of the population only speak Spanish, whereas 38% use Spanish and Catalan (see survey Knowledge and social use of Valencian language, 2010: http://www.ceice.gva.es/va/web/dgplgm/enquestes). Because these two Romance languages are similar, the same survey showed that 90% of Spanish monolinguals understand Catalan. This percentage reached 100% in our sample. This information was obtained from the interview when monolinguals self-reported an acceptable or good comprehension of Catalan, but poor or null fluency. Thus, an important consideration in our study is that, for simplicity, we use the term monolingual throughout our work to refer to individuals in our sample who only speak Spanish. However, within a more comprehensive categorization, these participants could be referred to as passive bilinguals because Spanish speakers who permanently reside in the Valencian region do not speak Catalan but are usually able to understand it. Finally, it is worth mentioning that our bilingual group did not include immigrant individuals. This rules out possible between-group differences in life conditions and cultural background, given that bilinguals and monolinguals in the Valencian region share the same sociocultural context and environment (e.g., neighborhood, school system, workplace).

Participants were invited to undergo a second magnetic resonance imaging (MRI) and neuropsychological evaluation, which took place between 6 and 9 months after the baseline acquisition. Fifty-nine participants (43 monolinguals and 16 bilinguals) performed this second evaluation. These 2 samples differed significantly on sex (*χ*^2^ = 6.68; *p* = 0.01) and were close to differing significantly on age (*t*_(57)_ = − 1.36; *p* = 0.09). These differences limit the interpretation of longitudinal analyses, given that any possible result could simply reflect baseline differences in these variables. Therefore, we used sequential matching [21, 35] to select a subsample of 16 monolinguals from the pool of 43 that were matched on sex (12 men and 4 women) and showed minimal differences in age (*t*_(30)_ = − 0.57; *p* > 0.1) and years of schooling (*t*_(30)_ = − 0.36; *p* > 0.1) compared to the bilingual sample. The mean time between the first and second MRI sessions in the selected sample of 32 participants was 6.91 ± 1.3 months. In the subsequent sections, all analyses referred to as cross-sectional involve the whole sample of 99 participants, whereas the analyses referred to as longitudinal involve the subsample of 32 participants. Longitudinal analysis with the whole unmatched sample of 59 participants yielded similar differences to those reported in the “Results” section. Furthermore, there were no significant differences between the whole sample and the follow-up sample in demographic variables, parenchymal volume, or any of the neuropsychological variables (all *p* > 0.1).

### Neuropsychological assessment

All participants underwent a structured clinical interview and a neuropsychological assessment, which included a short form of the Boston Naming Test [36], a Word List Acquisition and Recall test, two fluency tests (semantic and phonetic), a remote memory test, and the clock-drawing test [37]. The results of these tests were transformed into *z*-scores and averaged, in order to obtain a composite measure of the global cognitive level. Participants also completed the Mini-Mental State Examination (MMSE) [38, 39] and the Functional Activities Questionnaire (FAQ) [40]. Statistics for neuropsychological tests are reported in Table 1. In addition to the memory impairment, 96% of the participants (94.9% of bilinguals and 96.7% of monolinguals) showed impairment in another cognitive domain, thus meeting the criteria for multiple-domain amnestic MCI.

**Table 1:** Sociodemographic and neuropsychological variables for monolingual and bilingual MCI patients

	Monolinguals (*N* = 60)^a^	Bilinguals (*N* = 39)^a^	Statistical differences	*p* value
Gender	M/F = 26/34	M/F = 24/15	*χ*^2^ = 3.13	0.08
Age	73.58 (5.76)	74.26 (5.78)	*t* = − 0.56	0.57
Years of schooling	8.62 (3.45)	8.33 (2.43)	*t* = 0.47	0.63
Cognitive level	− 0.05 (0.6)	0.07 (0.64)	*t* = − 1.00	0.32
MMSE	26.95 (2.63)	27.23 (2.18)	− 0.55	0.58
FAQ	3.3 (2.58)	3.82 (2.48)	− 0.97	0.32
Boston	9.33 (1.45)	9.77 (1.31)	− 1.52	0.13
Phonetic fluency	8.37 (2.14)	8.51 (2.62)	− 0.30	0.76
Semantic fluency	10.63 (2.47)	10.74 (2.19)	− 0.23	0.82
WLA	9.03 (2.88)	9.79 (2.78)	− 1.30	0.20
WLR	1.07 (0.86)	1.10 (0.91)	− 0.20	0.84
Remote memory	9.18 (1.46)	9.49 (1.23)	− 1.08	0.28
Clock-drawing	7.14 (1.80)	7.00 (1.41)	0.39	0.69

*N* sample size, *M/F* males/females, *χ*^*2*^ chi-squared test, *t t*-value for two-sample *t* test, *MMSE* Mini-Mental State Examination, *FAQ* Functional Activities Questionnaire, *WLA* word list acquisition, *WLR* word list recall

^a^Mean and standard deviation (in parentheses) are shown for quantitative variables

### MRI acquisition

MRI data acquisition was performed on a 3T MRI scanner (Siemens Magnetom Trio, Erlangen, Germany) using a 12-channel head coil. Participants were placed inside the scanner in the supine position, and their heads were immobilized with cushions. Whole-brain 3D images were collected using sagittal T1-weighted images (MP-RAGE sequence, 176 slices, 256 × 256 matrix, TR = 2300 ms, TE = 2.98 ms, flip angle = 9°, spatial resolution 1 × 1 × 1 mm).

### Image preprocessing and statistical analyses

#### Global tissue differences

All analyses were performed with CAT12 (Computational Anatomy Toolbox; C. Gaser, Jena University Hospital, Jena, Germany; http://dbm.neuro.uni-jena.de/cat/) as implemented in SPM12 (Statistical Parametric Mapping 12; Wellcome Trust Centre for Neuroimaging, University College, London, UK; http://www.fil.ion.ucl.ac.uk/spm/) and SPSS 25 (IBM Corp.). Before data processing, the first quality check was conducted to detect images affected by important inhomogeneity or movement artifacts. To study global tissue differences, individual volumes of gray matter (GM), white matter, and cerebrospinal fluid were estimated after applying the standard segmentation procedure implemented in CAT12. Then, the brain parenchyma volume for each participant was obtained from the sum of the absolute volumes of gray matter and white matter. Between-group comparisons were carried out by means of ANCOVA models. For cross-sectional analyses, the model included global parenchymal volumes as dependent variable and bilingualism as independent variable. The possible confound of total intracranial volume (TIV) was included as covariate of no interest. For the study of longitudinal differences, the model included the parenchymal volumes for the first and second MRI scan as dependent variable, time as within-subject factor, and bilingualism as between-subject factor. The possible confound of time between scans (in months) and the differences in TIV estimations between the first and second MRI scans were included as covariates of no interest. Planned comparisons were evaluated to test the hypothesis of higher atrophy in bilinguals in the cross-sectional analysis and the hypothesis of a higher rate of atrophy in monolinguals in the longitudinal analysis, using a significance level threshold of *p* < 0.05.

#### Region-based morphometry

In order to study region-specific volumetric differences, region of interest (ROI) analysis implemented in CAT12 was performed. In this analysis, also called region-based morphometry (RBM), an anatomical atlas is transformed into native subject space, and the sum of the local GM inside the atlas’ pre-defined ROIs is estimated. The LONI Probabilistic Brain Atlas (LPBA40) [41] was used as a reference atlas. In this atlas, the whole brain is divided into 56 parcels comprising both cortical and subcortical areas. Statistical analyses were similar to the ones used to investigate global tissue differences. For cross-sectional analyses, ANCOVA models were performed, including ROI volumes as dependent variable, bilingualism as independent variable, and TIV as confound. For longitudinal analyses, ANCOVA models were carried out, including ROI volumes for the first and second MRI scans as dependent variable, time as within-subject factor, and bilingualism as between-subject factor. Again, the time between scans (in months) and differences in TIV estimations were included as confounds. Then, planned comparisons were conducted to test the hypothesis of higher atrophy in bilinguals in the cross-sectional analysis and a higher rate of atrophy in monolinguals in the longitudinal analysis, using a significance level threshold of *p* < 0.05 false discovery rating (FDR) corrected.

## Results

### Cross-sectional sociodemographic and neuropsychological results

**Fig. 1:**
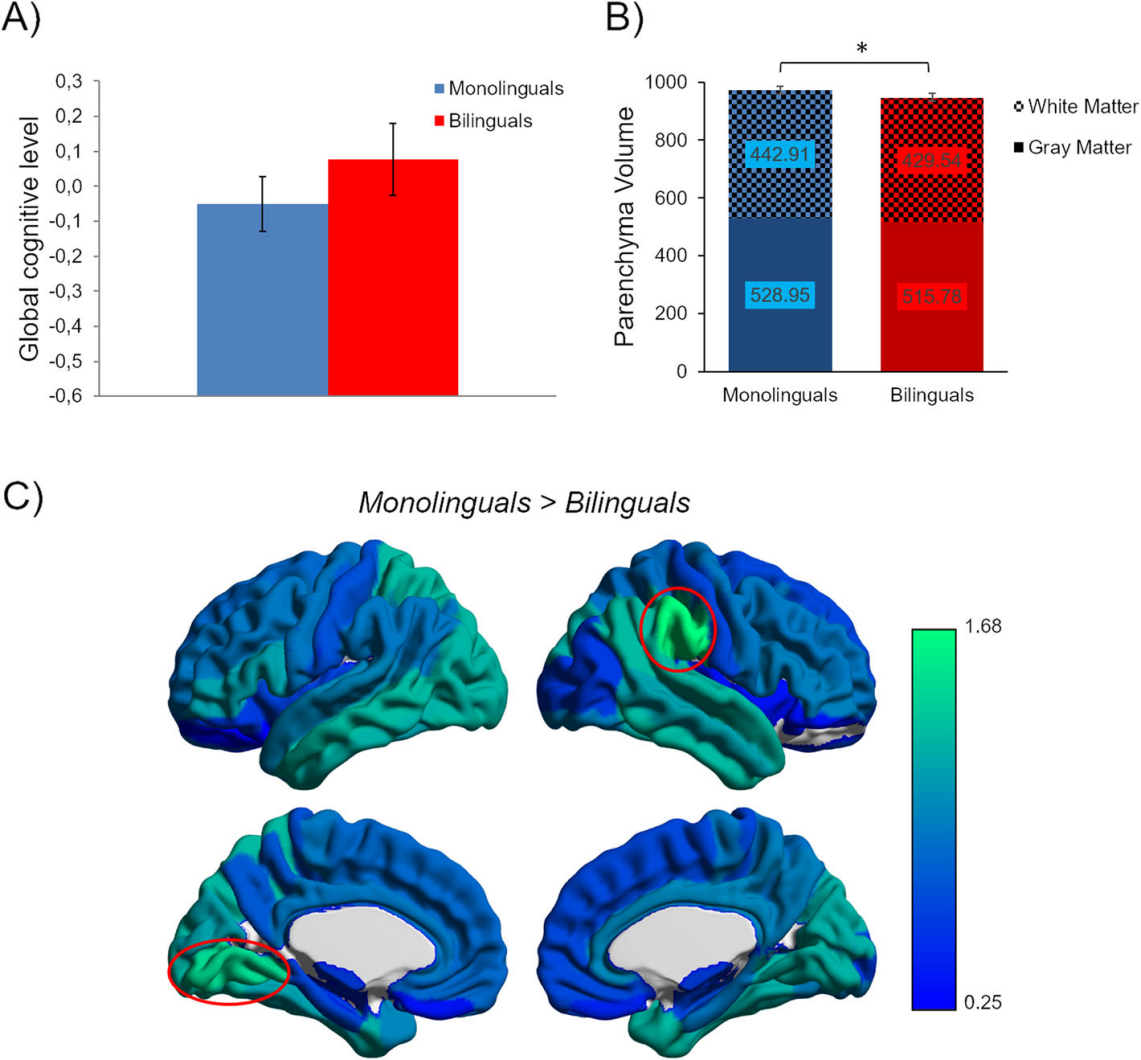
Cross-sectional results. **a** Mean and standard error bars for global cognitive level measure. **b** Mean and standard error bars for parenchyma volume (cm^3^). The graph is stratified in order to show the contribution of gray matter and white matter. *Significant differences at a threshold of *p* < 0.05. **c** Region-based morphometry results. The figure shows the brain parcels of the LPBA40 atlas. Red circles show the areas with significant gray matter volume reduction in bilinguals compared to monolinguals (*p* < 0.05 FDR corrected). The color bar represents the corrected log-scale *p* value FDR applicable to each parcel

We used *t* tests or chi-squares to compare these variables (see Table 1 and Fig. 1a). The results confirmed that there were no significant between-group differences between the bilingual and monolingual groups in age, gender, years of schooling, and global cognitive level. Importantly, the two groups did not differ in their performance on the Boston Naming Test in Spanish or in the analyses of the other neuropsychological tests. This pattern of results confirmed that the two groups presented a similar cognitive status at the time of the first MRI scan.

### Cross-sectional MRI results

In agreement with our hypothesis, the study of global differences in brain parenchyma volume showed reduced brain volume (*t*_(96)_ = 3; *p* = 0.002) in bilinguals, compared to monolinguals (see Fig. 1b). We ran an RBM analysis to locate where these differences were more prominent (see Fig. 1c). This analysis showed that bilinguals, compared to monolinguals, present significantly lower volume in the right supramarginal gyrus (*t*_(96)_ = 3.48; *p* = 0.021 FDR corrected) and the left lingual gyrus (*t*_(96)_ = 3.12; *p* = 0.034 FDR corrected). The opposite comparison (bilinguals > monolinguals) did not show any significant differences, even when using a lower threshold of *p* < 0.05 uncorrected.

### Longitudinal neuropsychological results

**Fig. 2:**
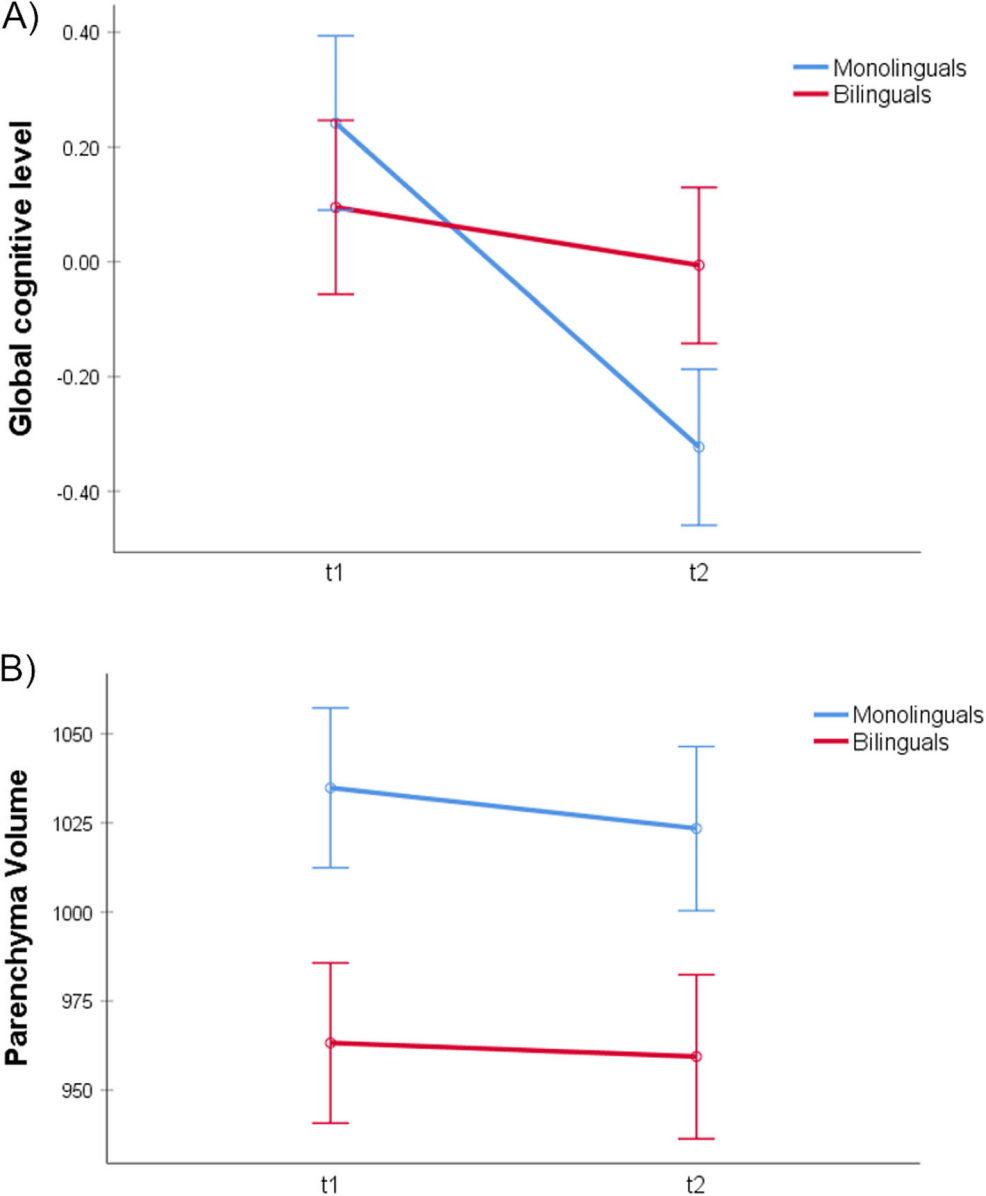
Longitudinal results. **a** Mean and standard error bars for global cognitive level measures at the time of the first and second neuropsychological evaluations. **b** Mean and standard error bars for parenchyma volumes (cm^3^) at the time of the first and second scans

Two participants (one bilingual and one monolingual) did not complete the second neuropsychological evaluation and were excluded from this analysis. Similar to the brain atrophy analyses, we used an ANCOVA model to study the hypothesis of higher cognitive decline in monolinguals than in bilinguals. Thus, the global cognitive level for the first and second evaluations was included as dependent variable, time as within-subject factor, bilingualism as between-subject factor, and time between explorations (in months) as covariate of no interest. Planned comparisons revealed a faster global cognitive decline in monolinguals compared to bilinguals (*t*_(27)_ = 2.50; *p* = 0.009; see Fig. 2a). Post hoc analyses investigating this effect showed that the global cognitive decline was significant in monolinguals (*t*_(14)_ = 4.02; *p* < 0.001), but not in bilinguals (*t*_(14)_ = 0.85; *p* = 0.205). The same analyses for individual tests showed a significantly faster decline in the monolingual group as compared to the bilingual group on the phonetic fluency test, that is, a similar pattern to the one observed in the global cognitive level (see Table 2).

**Table 2:** Neuropsychological results for the subgroups of monolinguals and bilinguals included in the longitudinal study

	Monolinguals		Bilinguals		Group effects	Time effects	Interaction effects
	Time 1	Time 2	Time 1	Time 2	*F*-score^1^	*F*-score^2^	*F*-score^3^
MMSE	26.8 (2.73)	25.7 (3.02)	27.7 (2.13)	27.1 (3.02)	1.46	5.87*	0.68
FAQ	3.80 (2.96)	5.40 (4.47)	3.07 (1.49)	5.40 (2.92)	0.15	9.15*	0.42
Boston	9.53 (1.68)	8.87 (2.36)	9.93 (1.03)	9.40 (1.12)	0.70	7.74*	0.09
Phonetic fluency	14.0 (8.34)	6.73 (2.79)	11.2 (3.73)	9.13 (1.81)	0.02	14.74*	4.19*
Semantic fluency	21.4 (16.3)	10.0 (3.05)	13.6 (5.41)	9.60 (1.55)	2.56	15.92*	3.45
WLA	10.0 (3.09)	9.73 (3.31)	8.47 (1.36)	9.13 (2.03)	1.74	0.75	0.90
WLR	0.87 (0.83)	0.53 (0.64)	1.00 (0.65)	1.13 (0.52)	3.39	0.49	2.51
Remote memory	8.80 (2.01)	9.13 (1.64)	9.47 (1.13)	9.93 (1.03)	2.71	1.54	0.06
Clock-drawing	7.50 (2.28)	6.29 (2.46)	7.40 (1.30)	6.73 (1.03)	0.08	9.39	0.73

Cells in the table indicate the groups’ means (standard deviation in parenthesis) for each neuropsychological test at time 1 and time 2. Two participants (1 bilingual and 1 monolingual) did not complete the neuropsychological evaluation at time 2. Thus, the table shows statistics excluding these participants (*N* = 30; 15 bilinguals and 15 monolinguals)

*MMSE* Mini-Mental State Examination, *FAQ* Functional Activities Questionnaire, *WLA* word list acquisition, *WLR* word list recall

*p < 0.05 uncorrected

^1^The main effect of bilingualism estimated by means of a two-way mixed ANOVA, including time as within-subject factor and bilingualism as between-subject factor

^2^The main effect of time estimated by means of a two-way mixed ANOVA, including time as within-subject factor and bilingualism as between-subject factor

^3^The interaction effect between time and bilingualism estimated by means of an ANCOVA model, including time as within-subject factor, bilingualism as between-subject factor, and time between explorations (in months) as covariate of no interest

### Longitudinal MRI results

As expected, planned comparisons to test the hypothesis of a higher rate of atrophy in monolinguals than bilinguals revealed a significant interaction between the time and language groups (*t*_(28)_ = 2.02; *p* = 0.027), reflecting a slower parenchymal volume loss in bilinguals compared to monolinguals across the time points (see Fig. 2b). Post hoc analyses investigating this effect showed that the parenchymal volume loss was significant in monolinguals (*t*_(14)_ = 4.75; *p* < 0.001), but not in bilinguals (*t*_(14)_ = 1.15; *p* = 0.134). The study of region-specific longitudinal differences by means of RBM analyses did not show any significant differences at a threshold of *p* < 0.05 FDR corrected in any ROI. However, using a more liberal threshold of *p* < 0.05 uncorrected, we found differences in the right cingulate gyrus (*t*_(28)_ = 2.46; *p* = 0.01), right putamen (*t*_(28)_ = 2.16; *p* = 0.019), right caudate (*t*_(28)_ = 1.74; *p* = 0.046), right hippocampus (*t*_(28)_ = 1.94; *p* = 0.031), and left fusiform gyrus (*t*_(28)_ = 1.93; *p* = 0.032). All these regions showed a similar pattern of faster volume reduction over time in monolinguals compared to bilinguals. Of note, these uncorrected results are reported for descriptive purposes to provide information about the areas within the parenchyma where the differences are more pronounced in our data, and they should not be used to draw inferences.

## Discussion

We investigated the neural bases of the putative protective effect of bilingualism against dementia by comparing the brain atrophy of bilinguals and monolinguals suffering from MCI. To this end, we selected two groups of monolingual and bilingual MCI patients with similar sociodemographic characteristics and education levels, living in the same area in the city of Valencia. The cross-sectional analysis showed that MCI bilinguals showed a greater amount of brain atrophy than MCI monolinguals, but no differences in global cognitive level or age. In the present study, we also took prospective longitudinal measures to shed light on atrophy rates in both groups. In agreement with our hypotheses, monolinguals showed higher brain atrophy rates and more cognitive decline than bilinguals in a 7-month period. Specifically, in this period, monolinguals, but not bilinguals, showed significant brain atrophy and cognitive decline. Together, our results suggest that the active use of two languages throughout life not only promotes CR, but also brain reserve, providing a neural-based framework that could explain why bilinguals, compared to monolinguals, show a delay in the onset of dementia.

The results of the present study are consistent with previous cross-sectional studies showing that bilinguals require a greater amount of neuropathology in the brain to manifest the same cognitive status level. Specifically, one study using positron emission tomography (PET) in patients with AD showed that bilinguals had lowered hypometabolism, especially in the temporo-parietal cortex [16], whereas another study using computed tomography showed more GM atrophy in bilingual AD patients than in monolingual patients [24]. Our cross-sectional analysis is consistent with all these results, but it presents some additional features that should be specifically discussed. First, our study is the first to demonstrate this neuroprotection in MCI patients. This condition is a preclinical form of dementia that preserves the ability to perform daily life activities. Our clinical sample was mostly composed of multi-domain amnestic MCI patients, and the results showed no age differences between the language groups because bilinguals were only 7 months older than monolinguals. These results were consistent with a previous report showing age differences in single-domain, but not multi-domain, MCI patients [14]. In that study, the authors proposed the possible coexistence of vascular risk factors as a possible explanation for the lack of age effects in this group, but this is unlikely in our sample because patients with vascular problems identified in the MRI were removed from the sample, and because bilingualism also protects against deterioration in stroke patients [42]. Thus, our data are the first to show increased CR in MCI, that is, higher atrophy in MCI bilinguals at the same cognitive level as MCI monolinguals. Second, we have demonstrated the effect of bilingualism on brain atrophy in the absence of relevant between-group differences in factors such as education, age, or the environment (i.e., the same city of residence), as obtained in previous studies [16, 24]. It is important that the two groups did not differ in their cognitive status, as assessed by a neuropsychological profile. Thus, the results obtained in the present study strengthen the interpretation of a bilingual advantage because we controlled for the potential confounding factors. According to recent proposals [9], differences may arise from a better capacity of bilinguals to functionally compensate for the greater loss of brain parenchyma. This increased neural compensation would arise from the continuous use of two languages, which entails a stronger use of certain brain areas involved in language control and executive functions [16]. The fact that our monolingual individuals could be better categorized as *passive bilinguals* favors this interpretation, given that the observed differences would not arise from the knowledge of the second language per se, but rather from the frequency of use.

RBM analysis revealed that the between-group significant differences shown in our cross-sectional study were mainly located in the lingual and supramarginal gyrus. These two regions have been reported to be affected by AD in functional and structural meta-analyses [43, 44]. Specific alterations in MCI individuals have been found in the lingual gyrus during tasks involving episodic memory [45]. Moreover, differences in the supramarginal gyrus have been shown when comparing anatomical likelihood estimate maps of MCI converters and non-converters in a meta-analysis integrating results from different neuroimaging modalities [46]. The right supramarginal area was also involved in previous morphometric studies in bilingualism. For instance, increased GM volume in this region has been related to better proficiency in the second language and the number of non-native languages spoken [47]. Furthermore, healthy older bilinguals showed higher white matter integrity than monolinguals in the superior longitudinal fasciculus [21, 22], that is, the white matter tract that connects the supramarginal gyrus to the frontal and temporal regions. All these results may suggest that the brain areas related to the use of language would increase their efficiency in bilinguals and, in turn, compensate for the effects of AD neuropathology.

One of the main objectives of this study was to investigate how bilingualism impacts the course of dementia. For this reason, we retested a subsample of patients who did not differ at baseline on any of the cognitive or sociodemographic variables (age, education, and gender). Our longitudinal results suggest that monolinguals have a faster rate of cognitive decline and brain atrophy than bilinguals. On the neuropsychological measures, both groups showed a significant cognitive decline on the MMSE, FAQ, phonetic and semantic fluency, Boston Naming Test, clock-drawing test, and the overall measure in the 7-month period. Crucially, monolinguals showed a greater decline than bilinguals on the overall cognitive measure. This result coincides with previous findings showing a heterogeneous pattern of cognitive decline that does not focus on any specific domain [48, 49]. In this regard, it is noteworthy that most of the patients who participated in this study were multiple-domain amnestic. Importantly, monolinguals, but not bilinguals, presented significant brain atrophy in the 7-month period. These results coincide with the findings from cross-sectional studies in healthy older individuals showing increased age-related brain atrophy in monolinguals compared to bilinguals [27–30]. In this regard, the results of our study agree with these findings and suggest that the neuroprotective effect of bilingualism is also maintained during the early stages of dementia. A possible neural mechanism driving this effect was proposed by Barulli and Stern, who suggested that neural compensation may increase brain reserve by promoting neuroplasticity [23]. Thus, the functional compensation required to maintain performance will eventually lead to changes in the brain itself. RBM analyses investigating the specific brain areas with a higher atrophy rate in monolinguals than in bilinguals did not show significant results at the pre-established threshold (*p* < 0.05 FDR corrected). However, uncorrected results suggest that the main differences found in the parenchymal analyses were located in areas related to language or executive control, such as the cingulate gyrus and the striatum [50], and in crucial areas in dementia, such as the hippocampus. Although speculative, we suggest that the continuous use of two languages in bilinguals may help to preserve the brain areas involved in controlling two languages and, potentially, executive control [51]. This neuroprotection would have a compensatory effect on the manifestation of cognitive symptoms of MCI and dementia [16].

This study has several limitations that should be considered. First, the sample in our study was composed only of MCI individuals. MCI cohorts are heterogeneous groups with variable rates of conversion to dementia. Therefore, cautious interpretation is required when extending the results to the protective effects of bilingualism on dementia. Second, due to participants’ drop out, only 59.6% of the initial sample performed the longitudinal recording. Furthermore, this subsample was unbalanced. Therefore, the longitudinal results were based on a relatively small sample of 32 individuals. However, the results obtained when comparing the unbalanced sample of 16 bilinguals and 43 bilinguals yielded similar results. Future studies with larger sample sizes may provide other differences not detected in our study. Third, the follow-up period could be considered short for a long-term disease such as AD. Therefore, our longitudinal analysis could be considered the first one to provide empirical evidence about short-term patterns of atrophy and cognitive decline in bilinguals and monolinguals with MCI. However, further studies investigating longitudinal changes in these populations using longer temporal windows are necessary. Fourth, the bilinguals in this study spoke Spanish and Catalan, which could be considered two similar languages. This could be a limitation in many studies of bilingualism; however, the protective influence of bilingualism on dementia has been demonstrated in different contexts (e.g., English-Spanish, English-Polish, English-Yiddish, Telugu-Hindi, Dutch-French). Furthermore, for the specific purposes of this study, this similarity might be a strength. It has been proposed that the relationship between cognitive reserve and bilingualism is due to the additional demands on the control system in bilinguals to effectively manage the two languages [8–10]. The positive findings in our study suggest that the contribution of bilingualism to CR is effective even in bilinguals speaking similar languages, where the cognitive demands on the control system might be considered lower than in dissimilar languages. In fact, in our study, we compared active and passive bilinguals; therefore, our results suggest that the contribution of bilingualism to cognitive reserve is related to the active use of the two languages and not just their comprehension. The same conclusions could be drawn from other studies [15].

## Conclusions

As a general conclusion, in this study, we found that bilinguals with the same cognitive level, age, years of schooling, sociocultural origin, and disease severity (MCI) as monolinguals showed lower parenchymal volume, especially in areas related to bilingualism and those previously described as affected in AD. Furthermore, monolinguals have prospectively shown a higher atrophy rate and greater cognitive decline than bilinguals over time. Together, our results suggest that bilingualism promotes both CR and brain reserve. The combination of these two factors may provide a neural framework to explain the nature and origin of the bilingual advantage in the delay of dementia.

## Data Availability

The datasets analyzed during the current study are not publicly available due to individual privacy protection but are available from the corresponding author on reasonable request.

## Abbreviations

AD: Alzheimer’s disease
CR: Cognitive reserve
MCI: Mild cognitive impairment
MRI: Magnetic resonance imaging
MMSE: Mini-Mental State Examination
FAQ: Functional Activities Questionnaire
GM: Gray matter
TIV: Total intracranial volume
ROI: Region of interest
RBM: Region-based morphometry
FDR: False discovery rating
PET: Positron emission tomography
